# Physiological changes of growth hormone during lactation in pup rats artificially reared

**DOI:** 10.1371/journal.pone.0220853

**Published:** 2019-08-13

**Authors:** Cesar G. Toriz, Angel I. Melo, Carmen Solano-Agama, Edgar Giovanhi Gómez-Domínguez, Ma. de los Angeles Martínez-Muñoz, Jorge Castañeda-Obeso, Eunice Vera-Aguilar, Elsa Liliana Aguirre-Benítez, Lucero Romero-Aguilar, Margarita González-del Pliego, Ismael Jiménez-Estrada, Maricela Luna, Juan Pablo Pardo, Javier Camacho, Maria Eugenia Mendoza-Garrido

**Affiliations:** 1 Departamento de Fisiología, Biofísica y Neurociencias, Centro de Investigación y de Estudios Avanzados del Instituto Politécnico Nacional (CINVESTAV-IPN), Mexico City, Mexico; 2 Centro de Investigación en Reproducción Animal, CINVESTAV-IPN ‐Universidad Autónoma de Tlaxcala, Ixtacuixtla, Tlaxcala, Mexico; 3 Departamento de Farmacología, CINVESTAV-IPN, Mexico City, Mexico; 4 Departamento de Embriología, Facultad de Medicina, Universidad Nacional Autónoma de México (UNAM), Mexico City, Mexico; 5 Departamento de Bioquímica, Facultad de Medicina, UNAM, Mexico City, Mexico; 6 Instituto de Neurobiología, UNAM, Neurobiología Celular y Molecular, Juriquilla, Queretaro, Mexico; Hospital Infantil Universitario Nino Jesus, SPAIN

## Abstract

During the lactation period, rat pups are fed by the dam, and the patterns of mother-pup interaction change during this period. Additionally, there are changes in feeding; first, mother´s milk is the only food needed for sustenance, and later, it is combined with solid food and water. GH serum concentrations depend on both maternal-pup interaction and energy metabolism. In the artificial rearing (AR) procedure, pups are deprived of mother-pup interaction, and the feeding pattern is controlled. This rearing paradigm has been used in rats to analyze the effects of maternal deprivation on social behavior. In the present study, we analyzed the variation in GH, acylated ghrelin and IGF-1 serum concentrations throughout the lactation period in AR pups. At pnd7, the maternal rearing (MR) pups responded to a 4 h fast with a drop in GH serum concentration, which is a well-known response to maternal deprivation. GH serum levels in the AR pups did not change, suggesting an adaptation phenomenon. A dopamine inhibitory effect of GH secretion was observed in pnd7 cultured somatotropes, suggesting dopamine regulation of GH secretion at this age. Acylated ghrelin serum levels in the AR pups showed an inverted pattern compared to that in the MR pups, which was related to the artificial feeding pattern. IGF-1 serum levels were lower in the AR pups than in MR pups, which was associated with hepatic GH resistance and with low *Igf1* mRNA expression at pnd7. Interestingly, at pnd14, both pup groups showed high hepatic *Igf1* mRNA expression but low IGF-1 serum levels, and this was inverted at pnd21. However, serum glucose levels were lower in the AR pups at pnd14 but reached the same levels as the MR pups at pnd21. Moreover, hepatomegaly and higher hepatic GH-receptor levels were observed in the AR pups at pnd21, which was in agreement with an absence of a solid food meal. During AR, the pups lost the maternal interaction-stimulated GH secretion, which correlated with lower IGF-1 serum levels during the first week of postnatal development. Later, the AR pups exhibited hepatic responses, in order to satisfy the metabolic demand for the normal weaning, with low carbohydrates levels in their meal.

## Introduction

At birth, growth hormone (GH)-expressing somatotropes are one of the most abundant types of pituitary secretory cells. Somatotropes-GH secretion is regulated by both central and peripheral components [1, 2]. Peripheral negative feedback is present with insulin-like growth factor 1 (IGF-1), which is principally a hepatic GH-stimulated hormone. At the central level, neurons from the arcuate nucleus secrete GH-releasing hormone (GHRH) and neurons from the periventricular nucleus secrete Somatostatin (SST) to portal vessels at the median eminence, reaching to the somatotrope cell. GHRH stimulates the synthesis and secretion of GH, and SST inhibits its secretion [3]. Peripheral components include acylated ghrelin, which is secreted by the pancreas very early in development, as well as by the stomach later [4]. This hormone directly stimulates the somatotrope cell-inducing GH secretion [5, 6]. Other peripheral components that are involved in GH secretion are sex steroids, free fatty acids and glucose [7, 8]. Because these last compounds are metabolically regulated by GH, this hormone is considered to be a metabolic hormone [8]. It is well known what the GH promoting growth actions are, most of which are through IGF-1 effects; however, many of its action are exerted directly in different tissues [9, 10, 11]. Nevertheless, GH effects on the body growth are not indispensable during prenatal development, but they are necessary for postnatal growth [12]. Postnatally, GH serum concentration varies during the lactation period: after birth, it is high throughout the first three postnatal days, followed by a decrease of approximately 50% during the next five days and a decline abruptly at day 14, and this hormone level is maintained without change until day 21 [13]. At the juvenile period, GH serum levels increase, and in the pubertal rat, hormone peaks are developed, reaching the highest postnatal levels [14].

In neonatal rats, there is an important regulator of GH serum levels, which is the mother-pup interaction pattern, which include the suckling behaviour of the pups and the nursing behaviour of the mother [15]. The neural mechanisms behind the regulation GH-secretion by the mother-pup interaction has not been elucidated, in our knowledge. It has been disclosed that vigorous tactile stimulus avoids the abrupt drop in GH serum levels observed in neonatal pups separated from the mother [16]. Furthermore, according to others authors [17], stimulus by the mother can be substitute by imitating the maternal licking with an artist´s brush. However, other researchers [18] conclude that maternal warmth is a critical component in the maternal modulation of GH. To the best of our knowledge, the mechanism involved in this pup response is not known. However, has been shown that the serum GH was higher in animals at pnd7 treated with haloperidol, with no changes in the pituitary hormone concentration [19]. This response to the dopamine DA D2 receptor (DA D2 receptor) antagonist was absent in rats at pnd14 [19]. Moreover, it has been shown that human somatotrope pituitary adenoma cells express D2 DA receptors, which together with somatostatin down-regulate GH secretion *in vitro* [20, 21]. However, another important component for development during lactation is milk. In addition to its nutritional components [22], milk has hormones and growth factors, which include GHRH [13]. However, feeding during lactation does not only include milk; around the middle of this period, pups initiate the ingestion of solid food and water. These diet variations require changes in the regulation of energy metabolism [23, 24]. Furthermore, the postnatal liver exhibits a metabolic development pattern related to the transition between the suckling and weaning period [25]. In the artificial rearing procedure (AR), the pup is deprived of the mother-pup interaction and the breast milk, and it is fed with a rat formula milk throughout the lactation period [26]. This procedure has been used to analyse the effects of maternal deprivation over social behaviour in rats [27, 28]. However, no studies have been performed analysing the endocrine system, specifically the GH in AR animals, especially because GH serum concentration is considered to be regulated by maternal-pup interaction and by the energy metabolism requirements during the different stages before weaning. In this study, we wondered how the GH serum concentrations are modified by the AR condition. To minimize the milk hormones and growth factor in MR pups [29] the experiments were performed after a 4h fasting period. Our results showed that MR and AR pups exhibited variations in GH, IGF-1 and acylated ghrelin serum concentrations throughout the lactating period. On this account, MR and AR pups differ markedly in their GH, IGF-1 and acylated ghrelin serum levels at pnd7, showing the role of maternal interaction and the feeding procedure. Later, at pnd14 and pnd21 the differences between both groups were related to the change in the type of food macronutrients, in MR pups, and the supplied of milk as the only source of carbohydrates. These differences showed the importance of nursing, involving the pup active suckling behavior and the maternal behavior, as well as the breast milk composition and the feeding transition during this dynamic development period.

## Materials and methods

### Animals

Wistar adult female rats (90 day-old) and their male offspring from the animal house facility of the Centre of Research and Advance Studies, Mexico were used. Animals were maintained at a constant room temperature (21 ± 1°C) and humidity (55%) with a dark/light cycle of 12 h, with the dark period beginning at 18:00 h, and they were given Purina Rat Chow and water *ad libitum*. All of the procedures described in this report were approved by the Cinvestav Animal Used and Care Ethical Committee (CICUAL, # 0267–05) following the Mexican Official Rule NOM-062-ZOO-1999 and the Guide from the National Institutes of Health (NIH-USA, #8023).

### Artificial rearing procedure

On the day of parturition (pnd0), the litters culled to eight pups. All procedures of rearing were developed in a small room (3x3x4 m) with controlled temperature (21 ± 1°C) and humidity (55%) with a dark/light cycle of 12:12 h, dark period beginning at 18:00 h. On pnd3.5, from three to four different litters, the males were taken and randomly assigned to be reared by a dam (mother reared-control; MR) or implanted with mouth cannula and raised artificially (AR). The litters size were 8 pups per litter in all groups of maternally reared pups. During the breeding process the MR pups and their foster dams were housed in clear Plexiglas cages (30 × 15 × 14 cm), with Purina Rat Chow and water available ad libitum, and left undisturbed except during bedding and cage change. For the AR group, the pups were selected randomly for artificial reared subject to a surgery, which consists in the implantation of a cannula through the cheek where milk is supplied [28, 30]. The surgery was performed according to previous works [27, 31]. Briefly, the pups received an implantation of a catheter (PE10 intramedic polyethylene tubing; Clay Adams Co. Parsippany NJ, USA) on their right cheek posterior to local anaesthesia with lidocaine (EMLA, USA. A guide wire (stainless steel, 0.25 mm in diameter, VWR), sheathed on one side with a Silastic tube (Dow Corning, VWR Scientific) and on the other with a PE10 catheter, was used to place the implant. The PE10 catheter was flared at one end with a flat washer to hold the tubing in place. Once the leader was dipped into reagent grade mineral oil (Sigma) to lubricate the tubing, it was led over the tongue and penetrated through the translucent cheek muscle and skin and gently pulled until the flared end made contact with the inner wall of the cheek. After that, the leader wire was removed and a dermic antibacterial cream (Neosporin) was applied topically at the site of penetration. Usually, the whole procedure lasted from 90–100 seconds. After the surgery, the AR pups were placed individually into a plastic cup (11 cm diameter, 20 cm tall, mounted on the AR system) whose bedding was a mix of wood chips (50% of clean and 50% of bedding change) and that it was changed during bedding and cage change of foster dams. The milk formula (Messer diet [32, 33]) was supplied by programmable Harvard infusion pump (PHD 22/2000, Harvard Apparatus, Holliston, MA, USA), which was programmed to infuse milk (Table 1) for 10 min every hour, 24h daily. The amount of milk that the pumps delivered was based on a fraction of the mean of the pups’ weight: at pnd3.5, the amount was 33% of the mean body weight and was increased 1% per day, up to a maximum of 45% of mean body weight. The pups were stimulated on their anus-genital region 2 times per day for 30–40 sec, with a warm, wet, camel hair paintbrush, to stimulate urination and defecation.

**Table 1:** Composition of macronutrients in artificial milk.

Macronutrient	g∙100 ml^-1^
Fat	10.9
Protein	7.5
Carbohydrate	7.6

Fat, protein and carbohydrate content in the milk prepared for the pups reared in an artificial condition.

### Additional sensory stimulus

The pups were stimulated with the paintbrush along the back with anterior-posterior movements for 45 sec, five times per day, daily until pnd21 of age [34]. Although this additional stimulation doesn´t mimics the maternal stimulation, it was showed that this is enough to prevent some effects of total maternal deprivation during early life, as alterations in the myelination and electrophysiological function of the sural nerve [30]. In order to avoid possible developmental alterations associated with the absence of maternal odours [35, 36], wood chips from mother-litters cage were used as an odoriferous stimulus. Moreover, the AR pups were in the same room as MR pups and their dams, which allowed them to hear the normal sounds of a litter. The animals were separated from the nest or from the cup 4 h before they were sacrificed (fasting period) at the ages of pnd7, pnd14 and pnd21. The AR pups were taken from their cup immediately after the 10 min of the milk infusion. A group of MR and AR pups of each age were immediately sacrificed after the separation from the nest or from the cup (no-fasted pups). However, in the case of MR animals they were with their siblings, with nest material and at 38°C, and the AR rats were altogether in the conditions as MR rats. The animals were sacrificed between 16:00 and 18:00 h by decapitation.

### GH, IGF-1, acylated ghrelin and glucose quantification

The GH concentrations were measured in the serum and in the culture medium from pituitary primary cell cultures by the method of Milliplex multi-analyte panel multiplex immunodetection kit with a CV 5.4% intra-essay, and 3% inter-assay (Millipore Corp, Billerica, MA, USA) in an equipment with Luminex x MAP technology (Magpix, Luminex Instruments, Millipore). Serum samples of 25 μl per pup were essayed. Total IGF-1 serum concentrations were measure by an IGF-1 mouse ELISA kit with a sensitivity of less than 4 pg∙ml^-1^ and a recovery of 98% (Abcam, Cambridge, UK). Serum samples were acid-ethanol extracted following the provider´s instructions. Ghrelin was measure as acylated ghrelin using the ELISA kit with a sensitivity of less than 1.56 pg∙ml^-1^, CV >8% intra-essay and <10% inter-essay according to the providers (MyBiosource, San Diego, CA, USA). Serum glucose was quantified using the equipment OneTouch UltraMini (Johnson & Johnson Medical Co., New Brunswick, NJ, USA).

### Secretion assay of cultured pituitary cells

To analyse the GH-secretion of pituitary cells from MR and AR pups, we used the experimental procedure of primary cell culture described by collaborators [37] with minor modifications. Cell cultures were performed with anterior pituitaries of MR and AR pups: at pnd7 (pools of 6 pituitaries, at pnd14 (pools of 3 pituitaries), and at pnd21pools of 2 pituitaries). Aliquots of 3 × 10^4^ cells were seeded in each well of a 48 multi-well (Costar, Corning Inc., Corning, NY, USA). Each well contained 1 ml of culture medium composed of medium 199 (Gibco, Carlsbad, CA, USA) and enriched with 10% (v/v) fetal bovine serum (Gibco). Cell cultures were maintained in standard conditions, and after 24h, the medium was replaced with 0.5 ml serum free medium and incubated for 60 min. A second 60-min incubation period was performed in the presence of 100 nM bromocriptine (Sigma-Aldrich Chem. Co, St. Louis, MO, USA). At the end of this time period, the medium was collected and stored at -20°C until it was assayed for GH. The cells were lysated, and the protein content was measured by the micro BCA assay (Thermo Sci., Rockford, IL, USA). GH values were normalized by the proportion of GH-positive cells present in the culture at each age.

### Real-time polymerase chain reaction for *Gh* and *Igf1*

Total RNA was extracted from homogenates of pituitaries and liver tissues from pups at the different ages studied using Tri reagent (Sigma) according to the instructions of the manufacturer. Total RNA (250 ng∙μl^-1^) was reverse-transcribed into complementary DNA (cDNA) by using Moloney murine leukemia virus reverse transcriptase (M-MulV, New England Biolabs, USA). *Gh* and *Igf1* expression were studied by real-time polymerase chain reaction (PCR) with 2 μl cDNA. With respect to *Gh*, the SYBR Green detection system and the Master Mix reagents kit (Qiagen, Hilden, Germany) was used, and for *Igf1*, the TaqMan detection system and the Universal PCR Master Mix reagents kit were used (Applied Biosystem, Life Technologies, USA). For the *Gh*, the primer used was developed by Dra. Maricela Luna [38]: Forward GGCCCAGCAGAGAACTGACAT; Reverse ATCAGAGCCTGGATGCCCTC. The following pre-developed assays from Life Technologies were used: for rat *Igf*1, the assay Rn00710306_m1, for *Gapdh*, which was used as a constitutive gene for *Igf1*, the assay Rn01775763_g1. For *Rn18s*, which was used as constitutive gene for *Gh*, the sequence name rRPS18qf was used: Forward TTCAGCACATCCTGCGAGTA; Reverse TTGGTGAGGTCAATGTCTGC (Sigma). Each cDNA samples was analyzed in triplicate and the corresponding no-reverse-transcriptase mRNA sample was included as a negative control. cDNA from adult male rats was used as a positive control, and the amplification ratio was obtained using the 2ΔΔ method. The DNA sequence of the PCR product of each sample was determined by the fluorescence-labeled dye-terminator reaction by using an ABI PRISM 310 Genetic Analyzer (Applied Biosystems). The PCR protocols were: 95°C for 10 min, 94°C for 15 s, 60°C for 30 s and 72°C for 30 s (40 cycles) for *Gh*, and 95°C for 15 s, 60°C for 60 s (40 cycles) for *Igf1*.

### Analysis of Glycogen and Triglycerides (TAGs)

Liver glycogen content was determined by the acid-hydrolysis method. After the hydrolysis of the hepatic glycogen with HCL, the glucose was converted into-glucose-6-phosphate by a hexokinase followed by its conversion into 6-phosphogluconic acid by glucose-6-phosphate dehydrogenase in the presence of NADP. In the reaction, NADP was reduced to NADPH which was measured spectrophotometrically [39]. The liver triglycerides content (TAGs) was determined by the quantification of glycerol released after the hydrolysis of the triglyceride molecules [40].

### Liver GH receptor quantification

To quantify the amount of GH receptor (GHR) in the liver of the MR and AR groups of pups, the following protocol was used: once we excised the liver of each individual, it was homogenized in RIPA buffer [150 mM NaCl, 50 mM Tris, 10% (v/v) glycerol, 0.1% (v/v) SDS, 1% (v/v), 23 mM deoxycocholic acid, 5 mM NaF, 2 mM Na_3_VO_4_, protease inhibitor cocktail (Cytoskeleton Inc., Denver, CO, USA) 4 mM PMSF, pH 7.4]. After sample protein quantification by bicinchoninic acid (BCA) protein assay (Pierce Thermofisher Sci., Rockford, IL, USA), aliquots of 45 μg of protein were resolved by 10% SDS-PAGE. The positive control samples were dissociated cells and membrane enrichment homogenate from adult rat liver cells, and the negative control sample was membrane enrichment homogenate of adult rat erythrocytes. The GH-receptor was determined by Western blotting using 1:1000 primary Polyclonal rabbit antibody against rat GH-receptor (MyBioSource, San Diego, CA, USA) and secondary antibody horseradish conjugated goat anti-rabbit antibody at 1:20,000 (Invitrogen). Actin was used as loading control, using a primary mouse monoclonal antibody (1:2000, donated by Dr. Manuel Hernandez, Cinvestav) and a secondary antibody goat anti-mouse (1:20,000, Invitrogen).

### Immunocytochemistry for GH and dopamine D2 receptor (DA D2 receptor)

Cultured cells obtained as mentioned above were stained for GH using an immuno-fluorescent assay. Aliquots of 3 × 10^4^ cells seeded over cover-glasses previously covered with poly-D-Lysine and culture in standard conditions. After 24 h in culture, the cells were fixed in 3.5% paraformaldehyde in PBS for 30 min, and permeabilised with 0.1% Triton X-100 for 10 min. Non-specific binding was blocked with 1% albumin-IgG free for 30 min, continued by the incubation with the antibodies: guinea pig anti-rGH (1:4000) and rabbit anti-Prl (1:10,000) obtained from Dr. Parlow (Pituitary Hormones & Antisera Ctr., Harbor-UCLA Med. Ctr., Torrance, CA, USA) and rabbit anti-hDA D2 receptor (1:1000) purchase from Millipore (AB5084P, Merck, NJ, USA). Incubation with primary antibodies was all night at 4°C, followed by a 60-min incubation with secondary antibodies: TRITC-donkey anti-guinea pig and TRITC-donkey anti-rabbit (1:400, Jackson ImmunoResearch, West Grove, PA, USA), and Alexa Fluor 488-donkey anti-rabbit (Life Technology, Eugene, OR, USA), for 2 h at room temperature. Cell nuclei were stained with DAPI (Vector, Lab. Inc., Burlingame, CA, USA). The stained cells were mounted with anti-fade medium (Vectashield, Vector). To define the hormonal cell type that expresses the DA D2 receptors, the cells were fixed and blocked a second time and processed to stain for GH or Prl. The GH-positive cell proportion in cultures, was obtained by counting the positive cells and the nucleus in an epifluorescence microscope (Observer.Z1, Zeiss, Göttingen, GE) with a 40× objective, and 10 fields were counted. Localization of the DA D2 receptor was visualised using a confocal microscope (TCS-SP 2, Leica, Germany) with an oil immersion 63x objective.

### Cytochemistry of liver tissue

The liver was extracted, weighed and fixed in 10% formaldehyde in PBS (v/v). Tissue was dehydrated in ethanol at different concentrations and embedded in paraffin and cut in 5–10 μm sections. The sections were stained with haematoxylin/eosin and were photographed in a light microscope (Axio observer.Z1 Zeiss, Jena, Germany) with a digital camera (AxioCam MRc, Zeiss), using the software Axio Vision real.4.8 (Zeiss) with an oil LCI plan-Neofluar 63x objective. After the image acquisition (double blinded), the cell area was measured using the ImageJ free software v1.47 (NIH, USA)

### Morphometric parameters of tibial bone

The right tibial bone was dissected from each pups of the MR and AR groups from the three different ages, and dried overnight at 38°C in an oven. The dried tibial bones were weighted using an analytical balance. The tibial bone length was obtained with a Vernier calliper and the lineal density was obtained as the relationship between dry weight (mg) and length (cm).

### Data analysis

All of the values are expressed as the mean ± S.E.M from at least 3 independent experiments. Statistical analysis searched for variations between the groups MR and AR, and the different ages were conducted by a parametric two-way ANOVA followed by a Tukey post-test when comparisons were performed between ages and Sidak when comparisons were performed between MR and AR. For the experiments with cultured cells, the analysis between groups, in each ages by two-way ANOV, because bromocriptine treatment showed opposite effects over GH basal secretion at pnd7 and pnd21. Unpaired Student´s *t*-test was carried out for differences in GH serum concentrations between pups immediately sacrificed and those sacrificed after fasting (GraphPad Prism 7, San Diego, CA, USA). Statistical differences between groups were considered to be significant at p< 0.05.

## Results

### Increase in body weight of MR and AR pups

**Fig 1:**
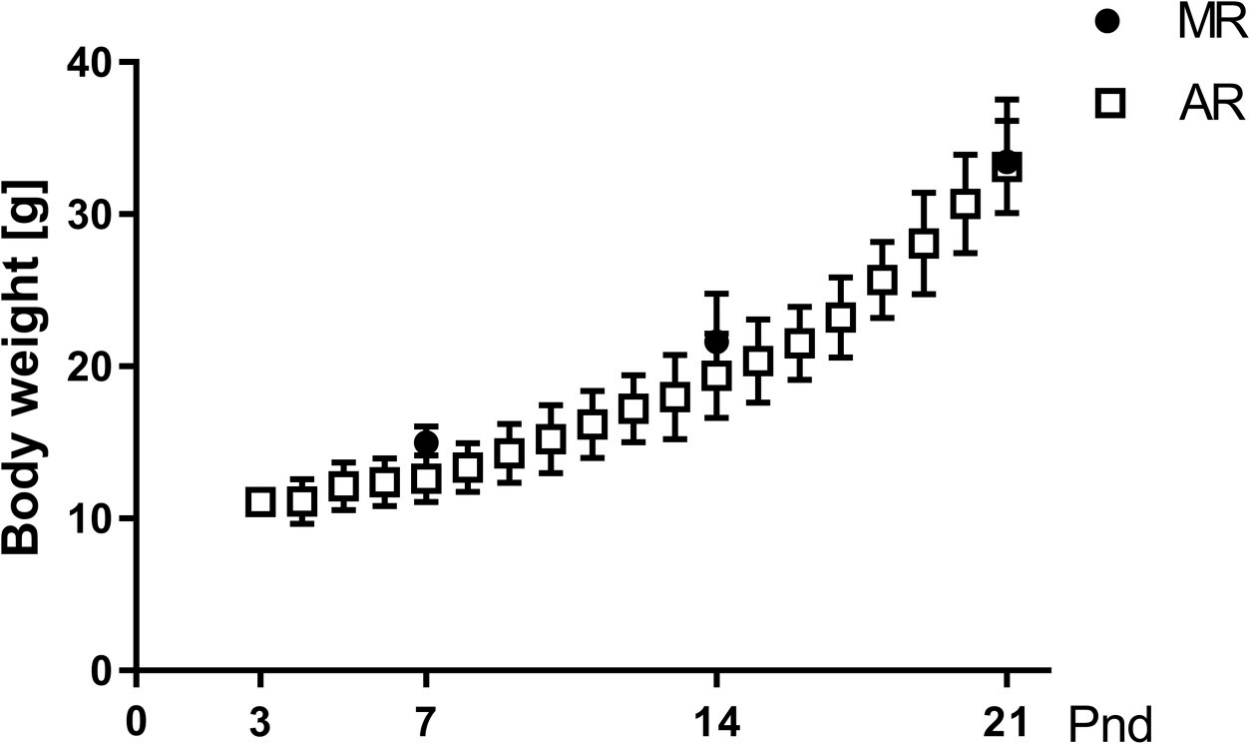
Body weight in MR and AR pups along the lactation period. MR pups (filled circles) were weight at pnd7, pnd14 and pnd21, while AR pups (open squares) were weight daily from pnd3 to pnd21. There were 53 MR pups for each age, and 202 AR pups during the lactation period.

The comparison of the body weight gain in MR and AR pups along the lactation period (Fig 1) did not show significant differences between both groups. Both pup groups showed a rate of body weight gain during the pnd14-pnd21 interval that was significantly higher than that during the pnd7-pnd14 interval (14.1 ± 0.56 and 7.0 ± 0.35 respectively).

### Serum GH concentrations in MR and AR pups

**Fig 2:**
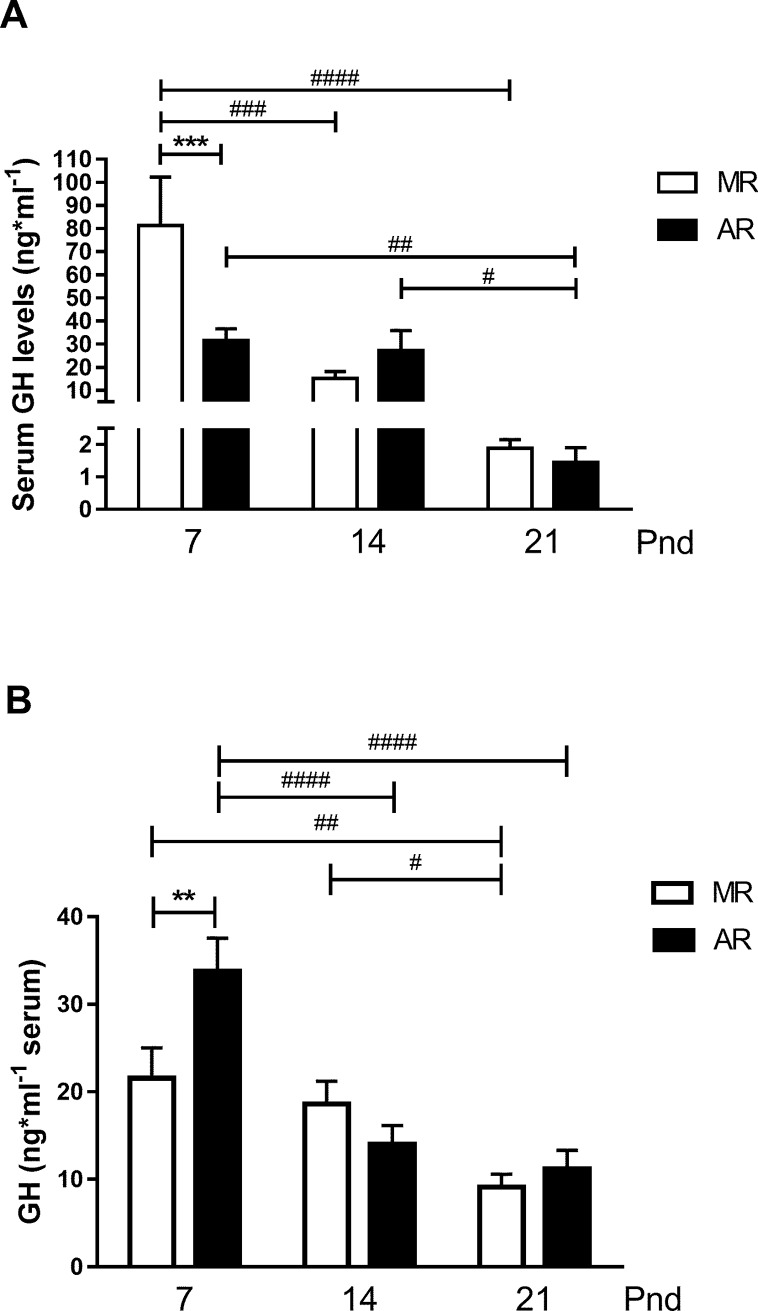
Serum GH concentration in pup rats at pnd7, pnd14 and pnd21. (A) GH serum concentrations of pup rats sacrificed immediately after they were removed from the nest or the cup, no-fasted pups (pnd7, MR = 6 and AR = 12; pnd14, MR = 9 and AR = 5; pnd21, MR = 8 and AR = 6), (B) GH serum concentrations of MR and AR pups after a 4h fasting period (pnd7, MR and AR n = 14; pnd14, MR n = 11 and AR = 12; pnd21, MR n = 16 and AR n = 15). Statistical analysis were performed for no-fasted pups (A) by one way-ANOVA, followed by Sydak´s multiple comparisons posttest for differences between ages in MR and AR pups, **#** P <0.05, **##** P <0.01, **###** P <0.001, **####** P < 0.0001, and for differences between MR and AR groups in each age *** P < 0.001., and (B) for fasted pups by 2 way-ANOVA, followed by Tukey´s multiple comparisons posttest for differences between ages in MR or AR groups **#** *P* <0.05, **##** *P* <0.01, **###** *P* <0.001, **####** *P* <0.0001, and for differences between MR and AR groups in each age ** *P* <0.01.

With the purpose of knowing the GH serum concentrations in pups with a minimum of disturbance, animals at pnd7, pnd14 and pnd21 were picked up from the nest and immediately sacrificed (no-fasted pups). Fig 2A shows that the higher serum GH levels were observed at pnd7 with a significant drop of 81% at pnd14, which continued at pnd21, when the values reached 2.3% of the GH serum concentration obtained in the pnd7 pups. In addition, serum GH concentration values from pnd21 pups were 87% lower than at pnd14. In the AR pup group, GH serum concentrations obtained from pups at pnd7 were quite similar than those from pups at pnd14. Differences in serum GH levels between MR and AR no-fasted pups were observed at pnd7, which the serum levels of AR pups were 61% lower than MR pups. However, at pnd21, a significant drop in the serum level of the hormone, 95%, was observed. To study the influence of maternal deprivation and a fasting period of 4h in circulating GH, we compared the serum levels of the hormone in MR pups with those from AR pups at pnd7, pnd14 and pnd21 (Fig 2B). The MR pups showed no changes in the GH levels at pnd7 and pnd14, but a 50% significant drop was observed at pnd21. AR pups showed a significant drop in the GH levels of 60% between pnd7 and pnd14, and no differences were observed between the second and third weeks of postnatal age. A comparison between the serum GH levels in both groups of pups disclosed differences at pnd7, while AR pups exhibited significantly higher levels than MR pups after the 4h fasting period (Fig 2B). However, when the GH circulating levels of the group of pups immediately sacrificed after their maternal separation was compared with those obtained from the MR pup group (after 4h fasting), a significantly higher value, 3.8 times more hormone, was observed in the first group (two way-ANOVA, followed by Sidak´s multiple comparisons pottest, *P*<0.0001). In contrast, at pnd21, the serum GH concentration was higher in the group of MR pups (after 4h fasting), 5.4 fold more (two way-ANOVA, followed by Sidak´s multiple comparisons pottest, *P*<0.0001). Interestingly, no differences in the serum GH levels between the two groups were observed at pnd14. At least, but very important, the circulating GH levels in AR pups immediately separated from the rear system were lower than those of the pups reared by the dam and immediately sacrificed, 34.2 ± 7.4 ng/ml and 81.3 ± 21.0 ng/ml, respectively (n = 6, unpaired *t* test, *P* <0.05).

### GH secretion in cultured pituitary cells from MR and AR pups and dopamine D2 receptor expression

**Fig 3:**
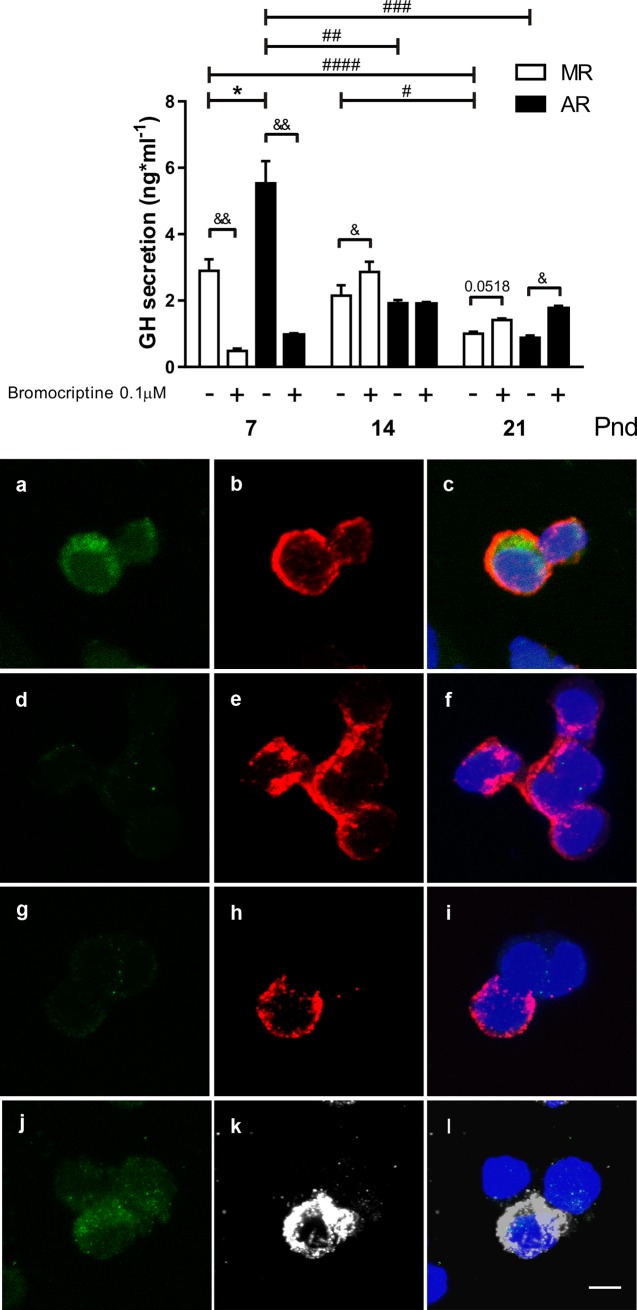
GH-secretion and dopamine D2 receptor localization in cultured pituitary cells from MR and AR pups at pnd7, pnd14 and pnd21. Cell cultures were performed with anterior pituitaries of MR and AR infantile rats: at pnd7 (pools of 6 pups), pnd14 (pools of 3 pups) and pnd21 (pools of 2 pups), n = 3 cell cultures. (A) Basal and bromocriptine-stimulated GH-secretion. Differences between GH basal and stimulated-secretion in MR and AR cell cultures at the three ages studied were compared by one way-ANOVA, followed by Sydak´s multiple comparisons posttest, for differences between ages, **#** *P* <0.05, **##** *P* <0.01, **###** *P* <0.001, **####** *P* <0.0001, for differences between MR and AR groups, * *P* <0.05, and for differences basal and bromocriptine stimulated, **&** P <0.05, **&&** P<0.01 (B) DA D2 receptor expression (green) in GH-positive cells (red) and Prolactin-positive cells (white) in pituitary cultures from pnd7 (a, b, c, j, k, l), pnd14 (d, e, f) and pnd21 (g, h, i) pups. Cultured pituitary cells were stained for DA D2 receptor (a, c, d, f, g, i, j, l), for GH (b, c, e, f, h, i) and for Prolactin (k, l). Bar = 15μm.

In cultures of pituitary cells from MR and AR pups at different ages, GH basal-secretion was observed with variations through the lactating period (Fig 3A). Cultured pituitary cells from MR pups at pnd7 and pnd14, showed no differences in GH basal secretion. In contrast, a significant decrease in GH basal-secretion was observed in the cell cultures from pnd21 pups with respect to those from pnd7 and pnd14, of 1.9 and 1.1 times less, respectively. Cultured pituitary cells from AR pups at pnd7 exhibited the highest GH basal-secretion, being 3.6 times more and 4.7 times more than those at pnd14 and pnd21, respectively. Although, no differences were observed in GH basal secretion in cultures from AR pups at pnd14 and pnd21. Comparing the basal-secretion of GH in cultured pituitary cells from MR and AR pups, a higher GH level was observed in cell cultures from AR pups at pnd7, 1.9 times more. When we considered that the somatotrope population changes during postnatal development [41], GH secretion by the cell cultures was related to the cell percentage of somatotropes for each age studied. The cell immunostained proportion was the number of GH-positive cells with respect with the total number of nuclei. No differences were observed between the two groups at the different postnatal ages. The mean values were the following: 48 ± 1% and 47 ± 1% at pnd7; 33 ± 2% and 30 ± 2% at pnd14; and 21 ± 1% and 23 ± 1% at pnd21, for the MR and AR pituitary cells, respectively. Basal GH secretion from MR pituitary cultured cells did not show differences along the rearing period (S1 Fig). However, the pituitary cell from AR pups showed higher GH basal secretion at pnd7 than at pnd14 and at pnd21 (2.9 and 2.0 times more). At pnd7, AR cultured cells showed a GH-basal secretion that was two times higher than those cells from MR pituitaries. No differences in the GH-basal secretion were observed for the other two ages. Bromocriptine treatment-induced changes depended on the age (Fig 3A). At pnd7, it reduced GH secretion in both MR and AR cultured cells. In contrast, at pnd14 and at pnd21, an increase in GH secretion was observed. In cultured pituitary cells from pnd7 pups, the DA D2 receptor was detected in 74 ± 3% of the GH-positive cells, and in cultured cells from 14pnd pups were 13 ± 2%, and was absent in these types of cells in pituitary cell cultures from pnd21 pups (Fig 3B). Prolactin-positive cells showed immunoreactivity for DA D2 receptors in pituitary cell cultures from pups at the three ages studied. In Fig 3B is shown Prolactin-positive cells and the expression of DA D2 receptor in cultured pituitary cells from pnd7 pups.

### Gh1 pituitary mRNA in MR and AR pups

**Fig 4:**
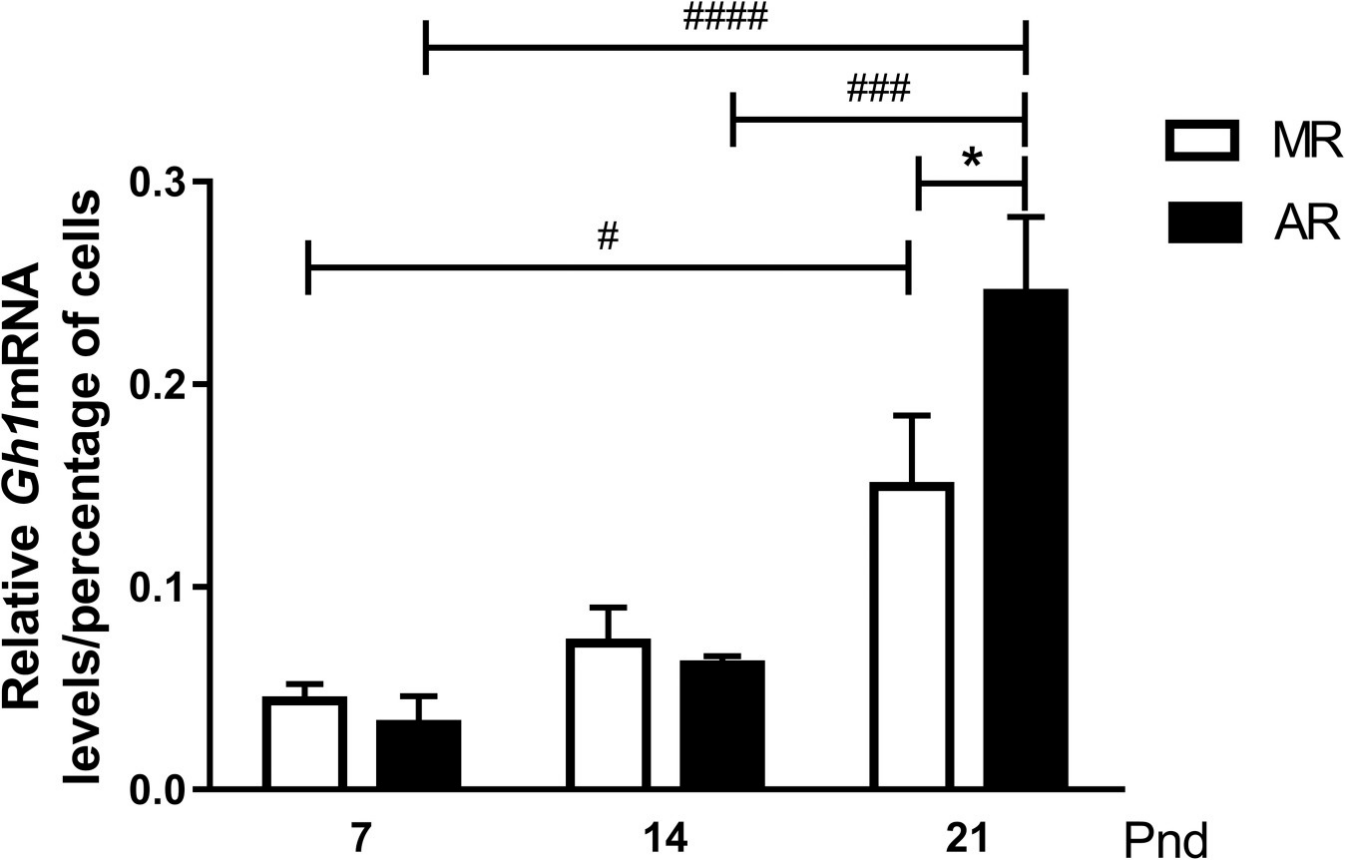
*Gh*mRNA in pituitaries from MR and AR pups at pnd7, pnd14 and pnd21. Total RNA was extracted from homogenates of pituitaries of pnd7 (pools of 8 pups), pnd14 (pools of 6 pups) and pnd21 (pools of 5 pups), n = 3 different extractions. *Gh*mRNA levels in pups were normalized according to male adult levels and fitted by the percent of somatotroph cells present in the pituitary at each age. Differences in pituitary content in MR and AR pups at the three ages studied were compared by two way-ANOVA, followed by a Tukey´s multiple comparisons posttest for differences between ages in both groups, **#** *P* <0.05, **###** *P* <0.001, **####** *P* <0.0001, and by Sidak´s multiple comparisons posttest for differences between MR and AR groups, * *P* <0.05.

The *Gh1* mRNA levels obtained from pup pituitaries were normalised with respect to male adult rat levels and, at the different ages, to the percent of GH-positive cells (Fig 4). No differences were observed in both pup groups at pnd7 and pnd14. At pnd21, a significant increase in the hormone mRNA was observed in both groups. For MR pituitaries, the difference of 3.4 times more was statistically significant between pnd7 and pnd21. In AR pituitaries, the hormone mRNA level at pnd21 was 7.7 and 4 times higher than at pnd7 and pnd14, respectively. The comparison of the *Gh1* expression at pnd21 in the two groups showed that AR pups had 1.7-fold more mRNA than the pituitaries from MR pups.

### Serum acylated ghrelin in MR and AR pups

**Fig 5:**
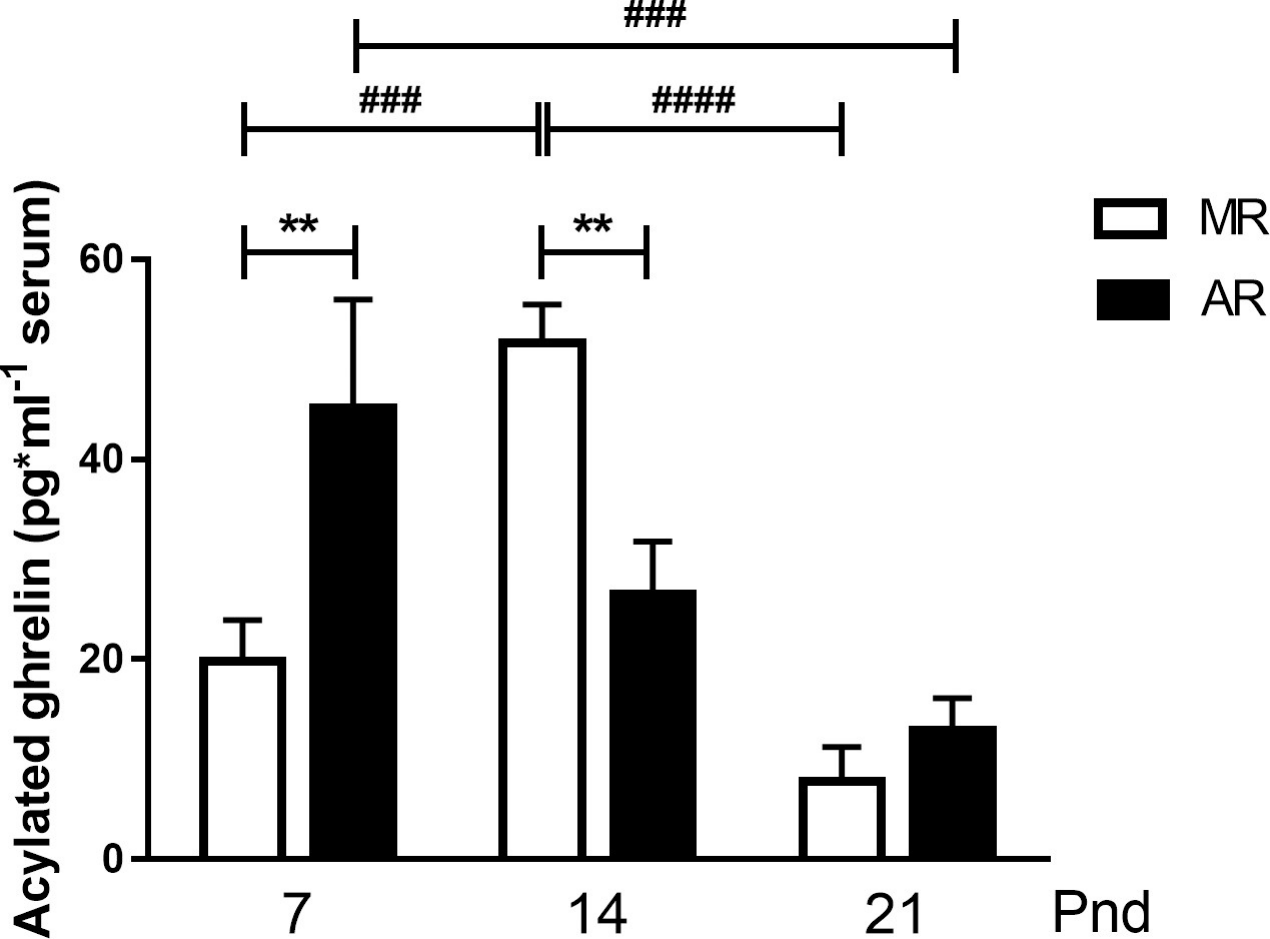
Acylated ghrelin in MR and AR pups at pnd7, pnd14 and pnd21 after a 4h fasting period. The hormone was measured in serum pools of 2 pups at pnd7 (MR n = 5 and AR n = 8, pools) and one pup for the other ages (pnd14, MR n = 5 and AR n = 6; pnd21, MR n = 9 and AR n = 7). Differences in serum acylated ghrelin concentrations were compared by 2 way-ANOVA, followed by Tukey´s multiple comparisons posttest for differences between ages in both groups, **###** *P* <0.001, **####** *P* <0.0001, by Sidak´s multiple comparisons posttest for differences between MR and AR groups, ** *P* <0.01.

Acylated ghrelin levels after fasting changed along the studied ages (Fig 5). After fasting, MR pups exhibited higher acylated ghrelin serum levels at pnd14 with respect to pnd7, 2.6-fold more, and pnd21, 6.6-fold more. However, in AR pups, the higher acylated ghrelin serum concentration after fasting was higher at pnd7 than at pnd21, with 3.5-fold more hormone. Comparing between groups, but at the same age, the circulating levels of acylated ghrelin was higher in AR pups than MR pups at pnd7, 2.3 times more, and in contrast, at pnd14, the higher levels were observed in MR pups, 2.0 times more. No difference in serum acylated ghrelin was observed between the two groups at pnd21 (Fig 5).

### Serum glucose concentration in MR and AR pups, in no-fasted and fasted condition

Table 2 shows the different serum concentrations of glucose in MR and AR pups at the three ages studied, and in a no-fasted or in a 4h fasted condition. In the MR pups, immediately separated from the nest, and in the AR pups, separated from the artificial rearing system and after finishing the milk infusion period, no statistic differences in serum glucose concentration were observed at the three ages studied. After fasting, the MR pups exhibited higher serum glucose concentrations at pnd14 and pnd21 with respect to pnd7 pups, approximately 1.9 times more. However, in the AR fasted pups the increase in serum glucose levels were observed until pnd21 pups, 1.9 and 1.6 times more than pnd7 and pnd14, respectively. Comparing between groups, at pnd21, the AR no-fasted pups exhibited higher serum glucose concentrations than MR no-fasted pups, 1.3 times more, and at pnd14, the MR fasted pups showed higher serum glucose levels than AR fasted pups, 1.4 times more. Finally, only in the MR pups, at pnd7, were observed a decrease in serum glucose levels after fasting, 1.8 times less.

**Table 2:** Serum glucose concentration in MR and AR pups, in a no-fasted or a fasted condition.

	Age (days)					
Groups	7		14		21	
	Serum glucose (mg*dl^-1^)					
	No-fasted	Fasted	No-fasted	Fasted	No-fasted	Fasted
**MR**	167 ± 11 &	94 ± 8 a, b, &	205 ± 10	174 ± 10 a, **	178 ± 6 **	174 ± 7 b
	n = 6	n = 8	n = 6	n = 12	n = 6	n = 9
**AR**	131 ± 36	104 ± 8 c	161 ± 12	116 ± 6 d, **	222 ± 5 **	199 ± 9 c, d
	n = 5	n = 6	n = 5	n = 13	n = 6	n = 9

Glucose was measured in individual samples. Differences between ages in the same group of pups were compared with one way-ANOVA followed by Sidak´s multiple comparisons posttest, a, b, c and d *P* <0.05. Differences between both groups of pups were compared with 2 way-ANOVA followed by a Tukey´s multiple comparisons posttest

** *P* <0.01. Difference between the same group, no-fasted *vs* fasted condition were compared with 2 way-ANOVA followed by a Tukey´s multiple comparisons posttest, & *P* <0.05.

### IGF-1 serum concentrations and bone growth in MR and AR pups

**Fig 6:**
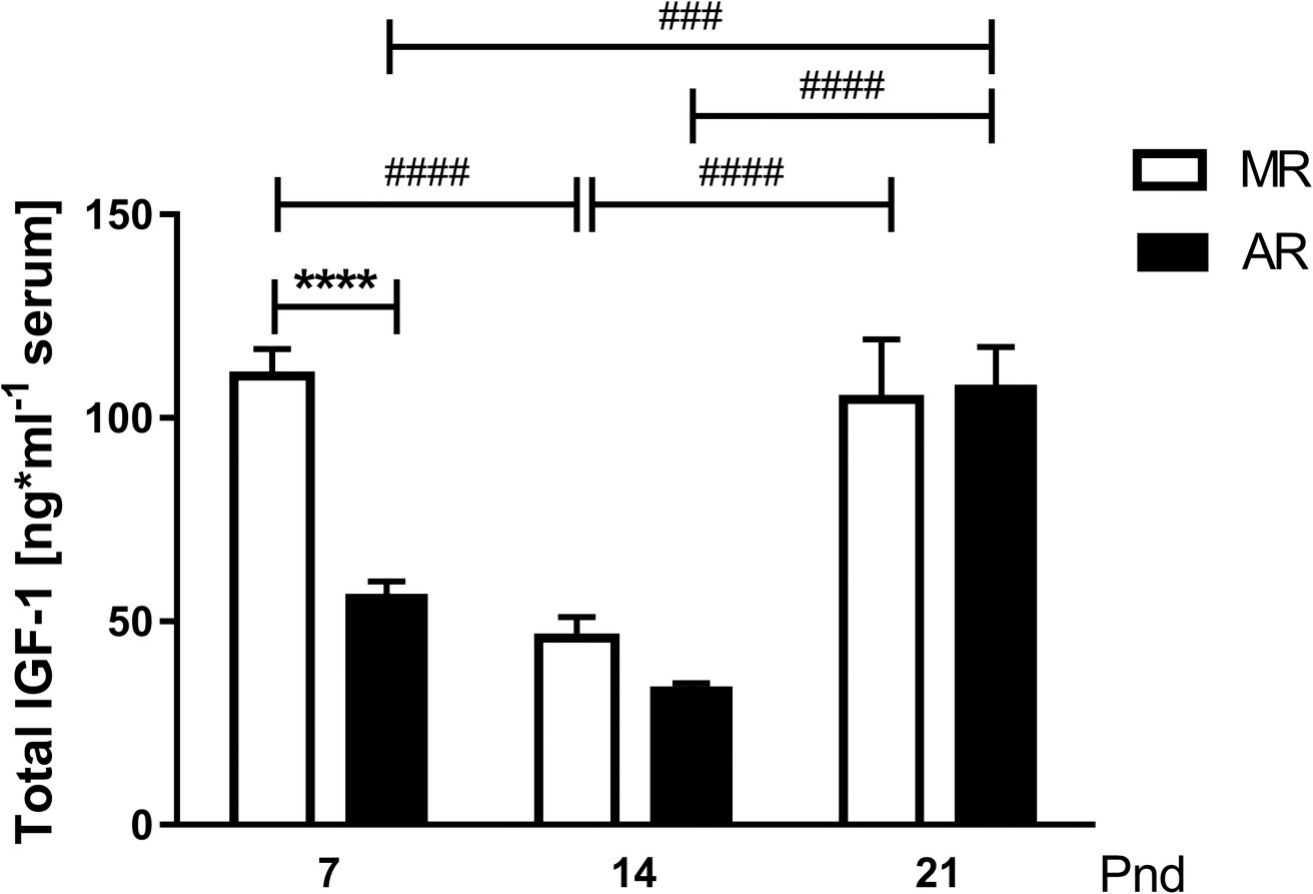
IGF1 serum concentrations in MR and AR pups at pnd7, pnd14 and pnd21. The growth factor was measured individually, n = 6. Differences in circulating IGF1 levels were compared by 2 way-ANOVA, followed by Tukey´s multiple comparisons posttest for differences between ages in both groups, **###** *P* <0.001, **####** *P* <0.0001, and by Sidak´s multiple comparisons posttest for differences between MR and AR groups, **** *P* <0.0001.

Because one of the GH actions is the stimulation of hepatic IGF-1 production, was analysed IGF-1 serum in 4 hour fasted MR and AR pups in search for a correlation between these hormones at the three ages studied. Fig 6 shows that the IGF-1 serum levels did not parallel those from GH in MR and AR pups (Fig 2). In MR pups, the serum IGF-1 concentrations at pnd14 were the lowest compared with at pnd7 and pnd21, approximately 2.3 times less. In AR pups, the IGF-1 serum levels at pnd7 did not differ from those at pnd14, but at pnd21, the levels were 1.9 and 3.3 times higher than at pnd7 and pnd14, respectively. A substantial difference in the IGF-1 circulating levels between MR and AR pups was observed at pnd7, where the MR pups were 2 times higher than the AR pups. When we considered the differences in the serum IGF-1 levels observed in MR and AR rats, we looked for differences in bone growth. The increase in the body weight and tibia length in the two groups of animals were quite similar, but the tibia lineal density was lower in AR rats than in MR rats, 18% at pnd7 and 16% at pnd14 (Table 3).

**Table 3:** Morphometric parameters of tibial bone in MR and AR pups at pnd7, pnd14 and pnd21.

	Age (day-old)	MR	AR
Length (cm)	p7	0.84 ± 0.01 n = 16	0.86 ± 0.02 n = 16
	P14	1.15 ± 0.02 n = 21, ####	1.10 ± 0.02 n = 20, ####
	p21	1.46 ± 0.02 n = 6, ####	1.48 ± 0.03 n = 6, ####
Tibia-bone weight (dry weight, g)	p7	6.91 ± 0.48 n = 16	5.84 ± 0.47 n = 16
	p14	10.80 ± 0.54 n = 21, ####	8.21 ± 0.63 n = 20, ####, **
	p21	20.20 ± 0.92 n = 6, ####	21.07 ± 0.91 n = 6, ####
Tibia-bone linear density (weight/length, g∙cm^-1^)	p7	8.18 ± 0.49 n = 16	6.74 ± 0.45 n = 16
	p14	9.30 ± 0.35 n = 21	7.79 ± 0.36 n = 20, *
	p21	13.80 ± 0.60 n = 6, ####	14.20 ± 0.53 n = 6, ####

The right tibial bone was obtained from the pups: at pnd7, MR and AR n = 16; at pnd14, MR and AR n = 21, and pnd21, MR and AR n = 6). Differences in: tibia length (cm), tibia dry weight (mg), and tibia linear density (mg∙cm^-1^) were analyzed by 2 way-ANOVA, followed by Tukey´s multiple comparisons posttest for differences between ages in both groups

**####** *P* <0.0001, and by Sidak´s multiple comparisons posttest for differences between MR and AR groups

** *P* < 0.01

* *P* < 0.05.

### Liver *Igf1mRNA* and GHR levels in MR and AR pups

**Fig 7:**
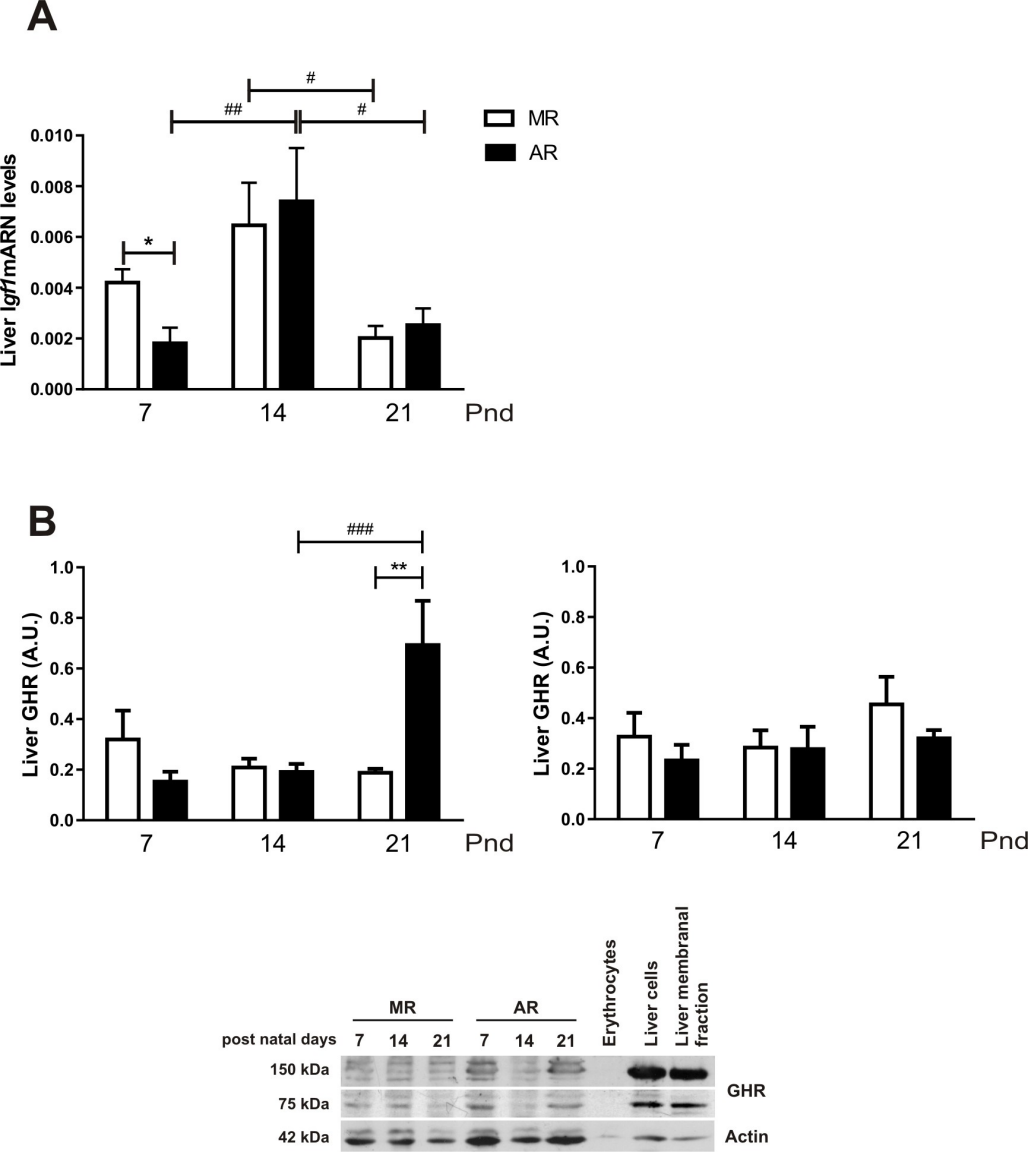
Liver *igf1*mRNA levels and GHR in MR and AR pups at pnd7, pnd14 and pnd21. Total mRNA and homogenates of individual livers of 4 hour fasted pups of the different groups and ages were obtained (pnd7, MR and AR n = 8; pnd14 and pnd21, MR and AR n = 6). *Igf1*mRNA and GHR levels in pups were normalized according to male adult levels. (A) Relative liver *igf1*mRNA levels of MR and AR pups. The values were analyzed by the 2 ΔΔ method using the adult values as control. Differences in the growth factor mRNA were compared by 2 way-ANOVA, followed by Tukey´s multiple comparisons posttest for differences between ages, **#** *P* <0.05, **##** *P* <0.01 and by Sidak´s multiple comparisons posttest for differences between MR and AR groups, * *P* <0.05. (B) Liver GHR levels, left panel corresponds to 75kDa receptor bands (monomeric) and right panel corresponds to 150kDa receptor bands (dimeric). Data correspond of individual samples, n = 6. Only in the monomeric receptor levels, statistical differences were obtained by 2 way-ANOVA, followed by Tukey´s multiple comparisons posttest for differences between ages in both groups, **##** *P* <0.01, **###** *P* <0.001, by Sidak´s multiple comparisons posttest for differences between MR and AR groups, ** *P* <0.01.

The main source of circulating IGF-1 is the liver; therefore, it was interesting to quantify liver *Igf1*mRNA from the MR and AR pup groups. As Fig 7A shows, for both groups, the liver *Igf1*mRNA expression profile fluctuates among the ages. In both pup groups, a higher expression of *Igf1*mRNA was observed at pnd14. However, in MR pups, it was only significant between pnd14 and pnd21 pups, being 3.3 times higher, while AR pups showed 5.7 and 3 times more *Igf1*mRNA expression at pnd14 than at pnd7 and pnd21, respectively. Comparing *Igf1*mRNA expression between MR and AR pups, a significant difference at pnd7 was observed, and the expression in MR livers was higher than in AR livers. Hepatic IGF-1 production requires the presence of GH receptor (GHR). No differences were observed in GHR, the monomer and the dimeric forms, in the MR pup livers (Fig 7B). In the AR pup livers, quantification of GHR showed similar values than those of MR pups at pnd7 and pnd14, but a significantly higher level in the monomeric form of the receptor was observed at pnd21.This difference was also found between MR pups at the same age

### Liver TAGs and glycogen content in MR and AR pups

**Fig 8:**
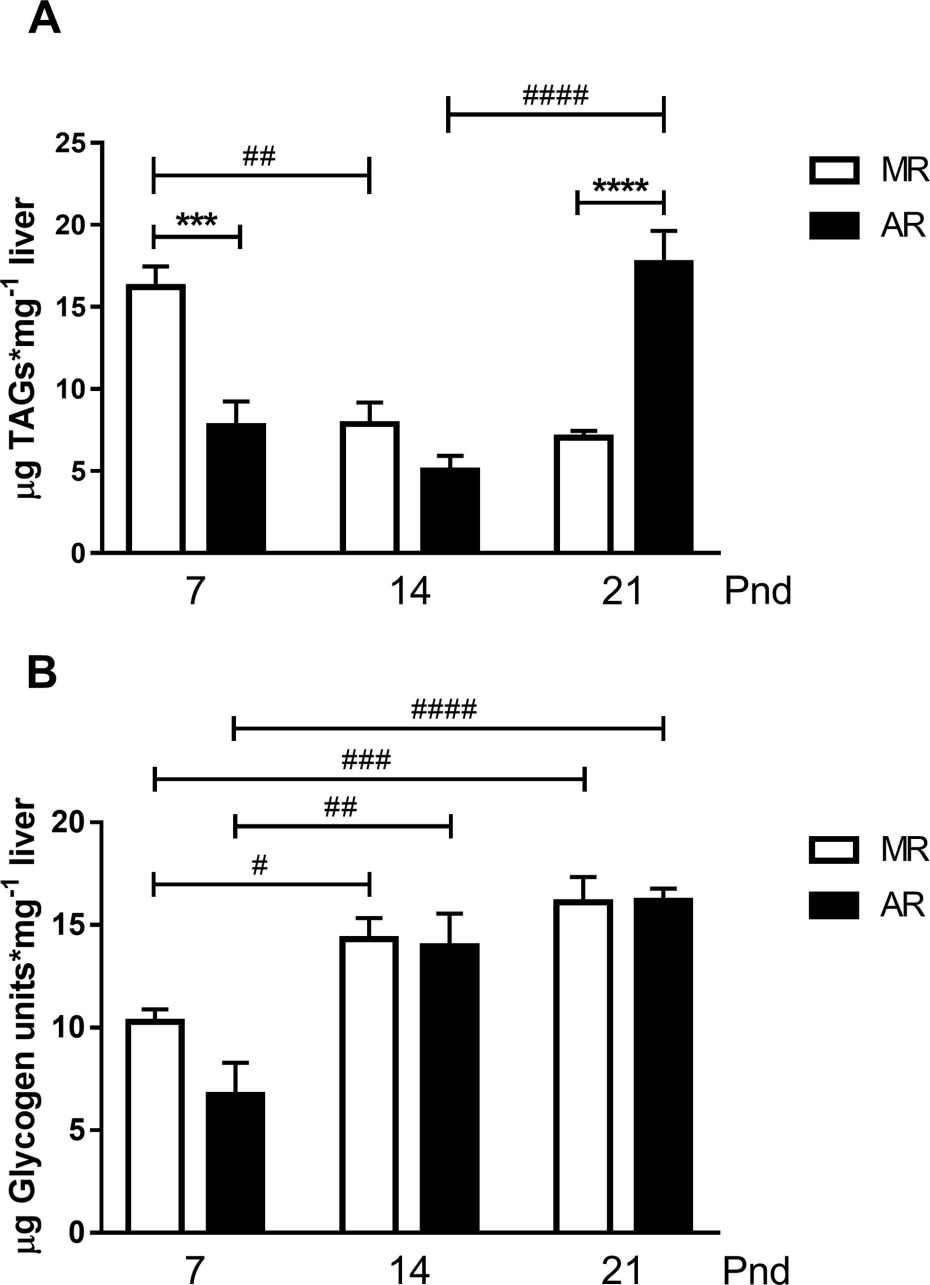
Liver triglycerides (TAGs) and glycogen content in MR and AR pups at pnd7, pnd14 and pnd21. Hepatic content of TAGs (A) and glycogen (B) of no-fasted pups were obtained. The measurements were performed in individual livers (n = 6, with one missed in AR group at pnd14). (A) Differences in liver TAGs content in MR and AR groups at different ages, (B) Differences in liver glycogen content in MR and AR groups at different ages. Differences in liver TAGs and glycogen content were compared by one way-ANOVA, followed by Sydak´s multiple comparisons posttest for differences between ages, **####** *P* <0.0001, **###** *P* <0.001, **##** *P* < 0.01, **#** *P* <0.05, and for differences between MR and AR groups, **** *P* <0.0001, *** *P* <0.001.

Fig 8 shows TAGs (A) and glycogen (B) content in livers from MR and AR pups no-fasted. The highest levels of liver TAGs in MR pups were obtained at pnd7, and at pnd14 and pnd21 the levels were around 50% less (Fig 8A). In contrast, in AR pups the highest liver TAGs content was at pnd21, being 2.3 times higher than levels at 7pnd and 3.5 times higher than at pnd14. When we compared the liver TAGs content of MR and AR pups at pnd7, a statistically significant difference was observed, AR pups had 2.1 times less content than MR pups. However, at pnd21 an inverse result was obtained, AR pups had 2.5 times more liver TAGs content than MR pups. In hepatic glycogen content (Fig 8B), the levels obtained in MR and AR pups were lower at pnd7 than at pnd14 and pnd21, 1.5 and 1.7 times less for MR group respectively, and 1.9 times less for AR group in both ages.

### Liver morphometric parameters in MR and AR pups

**Fig 9:**
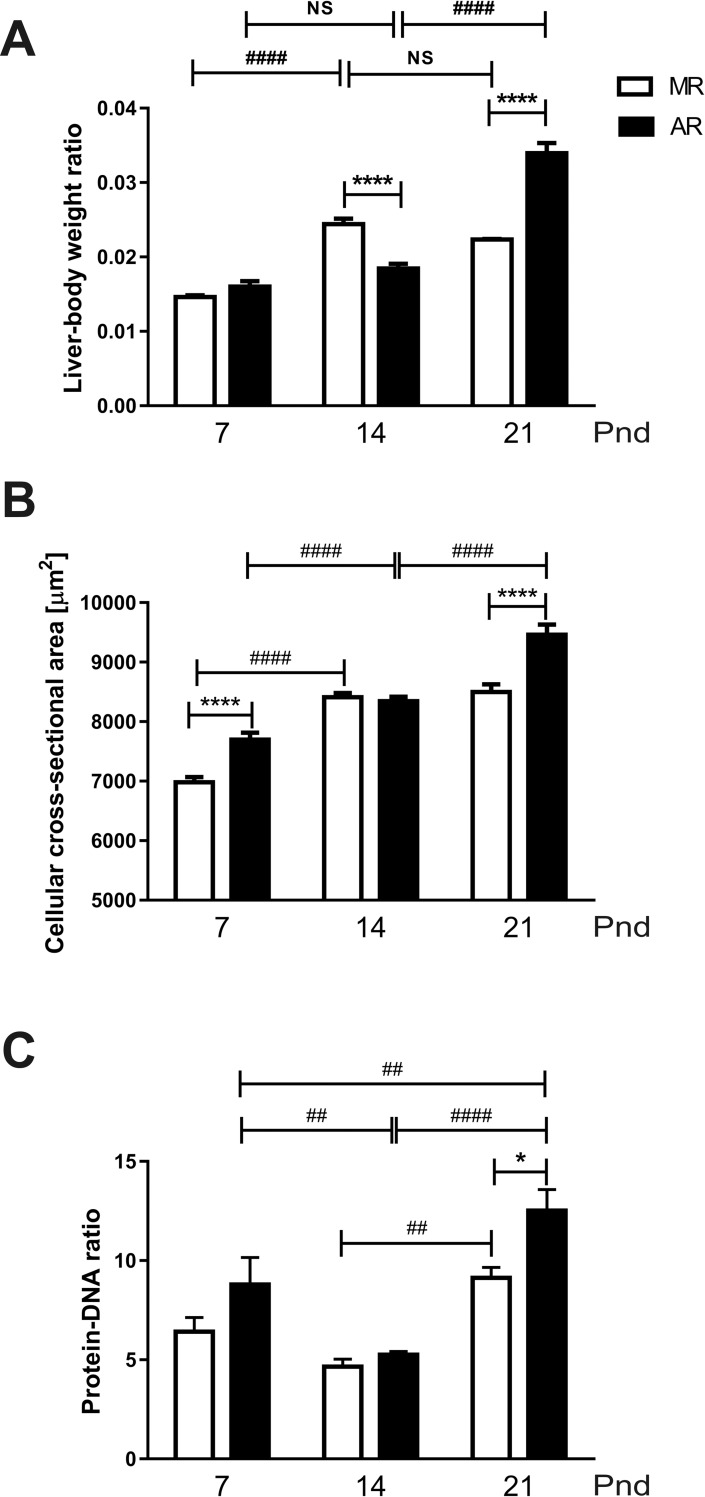
Liver tissue and hepatocytes parameters in MR and AR pups at pnd7, pnd14 and pnd21 pups. The tissue liver samples were obtained from the pups: at pnd7, MR and AR n = 8; at pnd14 and pnd21, MR and AR n = 6. (A) Differences in liver weight with respect to body weight of MR and AR pups at the different ages studied. (B) Differences in hepatocyte cell area in MR and AR groups. (C) Differences in protein: DNA ratios of hepatocytes from MR and AR pups. Differences between groups were analyzed by 2 way-ANOVA, followed by Tukey´s multiple comparisons posttest for differences between ages in both groups, **####** *P* <0.0001, **##** *P* <0.01, and by Sidak´s multiple comparisons posttest for differences between MR and AR groups, **** *P* <0.0001, * *P* <0.05.

The differences found in GHR and IGF-1 thus far could be due to changes in the liver physiology that can be reflected in morphometric parameters. Therefore, we measured the liver weight (as a fraction of the total body weight), the hepatic cell area (in μm^2^), and the protein ratio (with respect to the total DNA) of the liver (Fig 9). In the MR group, the liver weight increased, 1.6 times, between 7pnd and 14pnd, and then, it stopped changing (Fig 9A). In the AR group, between pnd7 and pnd14, no difference in the net increase of the liver weight was observed, but at pnd21, a significant increase was observed, approximately 2 times more (Fig 9A). When the liver weight was compared between MR and AR pups, a difference was observes: at pnd14, MR livers weighed more than AR livers, and the opposite was observed at pnd21 (Fig 9A). On the other hand, the mean cell area showed a constant increase between 7pnd and 14pnd, which was statistically significant (Fig 9B). For the MR group, the mean cell area at pnd21 was similar to that at pnd14. In the AR group, a higher mean cell area was obtained at pnd21 with respect to that obtained at pnd14, showing a 13% increase. Finally, the protein content, as a fraction of the total DNA in the hepatic tissue homogenates, was evaluated (Fig 9C). As with other measurements, there were oscillations in both the MR and AR pups. The protein content had its lowest value at 14pnd, and then, it increased at 21pnd in both groups. A difference in the MR livers was observed at pnd21 with respect to pnd14, which was 1.9 times higher. In AR livers at pnd14, the liver showed less protein content than at pnd7 and pnd14, which was 1.6 and 2.4 times higher, respectively. The MR and AR livers exhibited differences in their protein content only at 21pnd, with the AR livers being 1.4 times higher than the MR livers (Fig 9C).

## Discussion

Our results showed that MR rats at pnd7 exhibit a depression in serum GH after maternal deprivation, which is a phenomenon described by others [16]. This response was present although MR pups were kept with their siblings and at 36°C [18], suggesting that the main factor is mother-pup interaction pattern. In the case of the AR pups at pnd7, no differences were observed in GH serum concentration between pups immediately removed from their warm nest and after a 4 h fasting period. We interpret these observations as a disappearance of the mother-pup interaction regulation system on GH-secretion, specifically related to the suckling behaviour [15, 16, 17, 18]. However, their GH serum concentrations were higher than those of MR pups after maternal deprivation but lower than those from pups that were immediately separated from their dams. Taking the hypothetical neural network that has been propose [15], GH-secretion of the AR pups is only regulated by the hypothalamic GHRH-somatostatin system. On the other hand, it has not been identified any neurotransmitter involved in the GH-serum concentration decrease after maternal deprivation. However, it has been observed that pup rats at pnd7, but not at pnd14, showed higher GH serum concentrations after haloperidol treatment suggesting the participation of dopamine D2 receptors [19]. Taking advantage of this information, we wondered if the infantile pituitary somatotrope cell expresses the DA D2 receptor and whether its stimulation inhibits GH secretion. We founded that in both MR and AR pituitary cell cultures, the addition of bromocriptine inhibits the spontaneous GH-secretion. The experiment on the culture cells from rats at pnd14 showed no bromocriptine response, suggesting that the D2 DA receptor in somatotropes is expressed transiently at very early postnatal development; its presence was corroborated by immunocytochemistry in GH-positive cells at pnd7. This finding allows us to involve a DA response as part of the direct inhibitory mechanisms in pituitary GH secretion at this age. However, no data is available about hypothalamic dopamine secretion after maternal separation and the relationship with GH serum levels. Furthermore, the fact that somatotropes from AR rats responded to bromocriptine but not to the separation from the nest suggests that they are adapted to the mother´s absence. However, the GH serum levels at pnd7 in AR pups immediately separated from the nest was lower than in the MR pups sacrificed immediately after maternal separation. This finding prompts us to believe that milk-GHRH could participate in GH secretion [13]. However, the lower serum concentrations in AR pups could be to the loose of the maternal interaction-stimulated GH-secretion [15]. Nevertheless, cultured pituitary cells from AR pups at pnd7 exhibited higher basal secretion than cells from MR pituitaries. Interestingly, no differences in the *Gh* mRNA expression were observed in MR and AR pituitaries. The differences between *in vitro* and serum GH levels observed in our study, suggest they correspond to the development of the hypothalamus-pituitary axis. According to earlier studies [42, 43, 44], hypothalamic somatostatin concentration increase with age during the lactation period and correlates with a decrease in GH serum levels, and an increase in pituitary GH content. It was concluded that neonatal rats are under a stimulatory hypothalamic control of GH secretion, exhibiting a pituitary insensitivity to somatostatin, in accordance to the high circulating levels of GH during this period. We interpreted both as an evidence of a deficit in maturity in the cell control of GH secretion. Later, at pnd14, the responses to maternal deprivation and to bromocriptine were not present. However, at pnd21, the serum GH concentrations were increased in both pup groups as a response to fasting, which shows the well-known GH response to this condition [3]. At this age, a stimulatory GH response to bromocriptine was observed, in either MR or AR pituitary cells. This bromocriptine response could be elicited by its partial agonist effect on the 5-HT1B and 5-HT2B receptors [45], which can stimulate the GH secretion [46, 47]. In GH secretion participates the hypothalamic regulators GHRH and SST, and the periphery hormones, acylated ghrelin and IGF-1. When we analysed the circulating levels of acylated ghrelin after fasting, we observed higher serum levels in AR pups than in MR at pnd7. It has been well documented that mouse at pnd7 responded to a 4-h fasting period, increasing their serum levels of acylated ghrelin [48]. However, the main source of acylated ghrelin is the pancreas at this postnatal age in rats [4], and it has been stablished that ghrelin cells responded to low glucose concentrations [49]. We observed that at pnd7, MR and AR pups exhibited low serum glucose levels after a 4 hour fasted period. Likewise, other researchers have found that rats at pnd6 after a fasting period of 4h are hypoglycaemic, with low serum insulin levels and high glucagon levels [24]. It is considered that during fasting, ghrelin is secreted in order to avoid a drop of the glycaemic concentration to a critical level [50], and in fact, ghrelin inhibits insulin secretion and stimulates glucagon secretion. The only nourishment that pups at pnd7 receive is the dam milk, which has low carbohydrate level [22]. During the first week of postnatal life, the dams stay at the nest 18h to 13h/day in a crouching posture [23], such that the pup can ingest different milk volumes during extended periods. In contrast, the AR pups were fed with a fixed cycle of 10 min of milk infusion and 50 min of fasting, which could elicit a higher acylated ghrelin response to fasting. However, the picture of the acylated ghrelin serum concentration after fasting was inverse in the two groups of animals at pnd14. We observed that fasted MR pups showed higher acylated ghrelin and serum glucose levels than at pnd7, and, in accordance to other researchers [43]. Until this age, most of the serum ghrelin origin it is from the stomach epithelium [4]. Pups at pnd14 suckled for shorter periods because the dam rests at the nest for less time and they search for food and water. It is known that the pups develop the satiety sensation at this age [23]. When we looked the stomach content of the pups, we observed, in addition to milk, solid food in the MR pups. A delay in the development of gastric ghrelin cells in lactating pups has been associated to an absence of solid food in the pup diet [51]. In this context, the low acylated ghrelin serum concentrations observed in AR pups could be due because these pups were fed only with milk. This interpretation is reinforced because the acylated ghrelin serum levels obtained after fasting in AR pups at pnd7 and pnd14 were very similar. At 21pnd the acylated ghrelin serum levels were quite similar in both pup groups, although AR pups their only food was milk. Taking into account that these pups had fixed periods of the meals, the levels of acylated ghrelin in AR pups could be associated to the periodicity of the milk infusion. Moreover, variations in serum ghrelin in response to cycles of feeding-fasting is a phenomenon that can be trained [50].

When we analysed the changes in IGF-1 serum concentration during the lactating period in the MR and AR pups, we observed differences related to the postnatal age of the animals. At pnd7, the circulating IGF-1 levels were high, and it decline with the age of the pups. However, pups of the group AR at pnd7 exhibited lower serum IGF-1, which correlates with lower GH serum levels. These results are in accordance to the well-known relation between the two hormones [52]. When we considered that most of the serum IGF-1 is provided by the GH-stimulated liver, we looked for the *Igf1*mRNA and GHR concentration in the liver OF FASTED PUPS. In AR pups, the hepatic *Igf1*mRNA was diminished as well as the serum concentrations, but the GHR in liver showed no differences with respect to the MR pups. GH serum levels were lower in AR than MR pups, and this correlates with *Igf1*mRNA levels. However, the IGF-1 serum levels in AR pups were significantly lower than in MR pups. It is known that GH hepatic signalling is inhibited by different factors as the suppressors of cytokine signalling (SOCS), which regulate the cytokine signalling [11]. It has been observed, in liver cells, that interleucine I inhibits the IGF-1 synthesis stimulated by GH, and has been postulated that the effect took place at the transcriptional level [53]. During the early development, gut epithelium is organised to function with regard to the nutritional requirements throughout the lactation period [54]. In mother´s milk, there are different growth factors present, as well as hormones, and inflammatory regulators, which regulate the intestinal cell proliferation and maturation [55, 56]. With regard to AR pups, it is necessary to consider the change at p3.5 to a synthetic milk, absent of bioactive milk factors, which could induce an inflammatory process, propitiating a drop in the *Igf1*mRNA, even with normal hepatic GHR expression. A decrease in the IGF-1 levels during the infantile period induces a decrease in the periosteal circumference and cortical thickness of large bones [57, 58]. In this context, in AR pups the less tibia linear density obtained, comparing with MR pups, correlates with low IGF-1 serum levels. Interestingly, the lowest levels of serum IGF-1 throughout the lactating period were obtained at pnd14, and they correlate with the drop in circulating GH. However, at this age, the liver showed an increase in growth with respect to the body weight, without any change in the GHR, and expressed higher amounts of *Igf1*mRNA. According to our observations, there is a negative control in the *Igf1*mRNA transduction and a stimulation cell hepatic proliferation. At the moment we do not know what is responsible for this phenomenon. Despite this limitation, regardless of the rearing type, at pnd21, the relation on the *Ifg1*mRNA/IGF-1 protein was inverted. In addition, the body weight showed a significant increment, where low transcript IGF-1 concentrations generate high protein concentrations. This finding could be explained with the acquisition of a mayor role of the GH-IGF-1 in the longitudinal growth after weaning. However, AR pups at this age showed hepatomegaly with hypertrophy cells and higher GHR concentrations. The explanation could be the lack of change in feeding in AR pups. Whereas the MR pups initiate their feeding with solid food and water at pnd14, the AR pups are fed only with milk. Rat milk has low carbohydrate content, 7.6%, while the standard rodent chow diet has 50.8% carbohydrate; then, the pups must change their metabolism to a diet rich in this macronutrient. Our observations in pups at this age showed that both groups of animals responded to fasting increasing their GH serum concentrations and they exhibited similar serum glucose levels. The measurement of liver TAGs content of AR pups at pnd21 showed higher levels than MR pups. Moreover, AR pups exhibited higher hepatic GHR concentration, and liver size than MR pups. One of the GH effects in liver is an induction of TGAs uptake [59]. In addition, glucose levels in AR pups were higher than MR pups, although their meal was carbohydrate low, suggesting an increase in GH-mediated induction of hepatic gluconeogenesis [60]. According to the liver glycogen content obtained in MR and AR pups at pnd21, an induction of glycogenolysis by GH is not the main effect. All these data suggest that AR pups develop hypertrophic hepatocytes with an increase in GHR to compensate for the low carbohydrate diet that they received. One of the GH effects in liver is an induction of glycogenolysis [59], and our results suggest that AR pups develop hypertrophic hepatocytes with an increase in GHR to compensate for the low carbohydrate diet that they received. According to this interpretation, the pup at the end of the lactating period matured to a metabolism that involved a diet rich in carbohydrates [24]. Our data showed that the variations in GH depend, at the neonatal stage, to mother-pup interaction, followed by the acquisition of macronutrients in the diet. This dynamic development is followed by GH, IGF-1 and acylated ghrelin changes that permit the pup maturation. The pups raised in an artificial condition must do the following: first, adapt to the absence of the maternal-pup interaction and the new type of milk that lacks the bioactive molecules; and second, respond to a metabolic demand with an invariable food during this period.

## Supporting information

S1 Fig GH-secretion normalized by the percent of GH-positive cells in cultured pituitary cells from MR and AR pups at pnd7, pnd14 and pnd21. Cell cultures were performed with anterior pituitaries of MR and AR pups: at pnd7 (pools of 6 pups), pnd14 (pools of 3 pups) and at pnd21 (pools of 2 pups), n = 3 cell cultures. Basal and bromocriptine-stimulated GH-secretion were normalized by the percent of GH-positive cells. Differences between basal and bromocriptine GH secretion were analysed by two-way ANOVA, followed by Sidak´s multiple comparisons posttest for differences between MR and AR groups, **####** *P* <0.0001. Statistical differences between MR and AR groups in GH secretion were analyzed by two way-ANOVA, followed by Sidak´s multiple comparisons posttest, * *P* <0.05, *** *P* <0.001, **** *P* <0.0001.
(TIF)
