# Supplementary figures and images for: Physiological changes of growth hormone during lactation in pup rats artificially reared
Source: PLoS One. 2019 Aug 13;14(8):e0220853. doi: 10.1371/journal.pone.0220853 (PMC6692037; doi:10.1371/journal.pone.0220853)

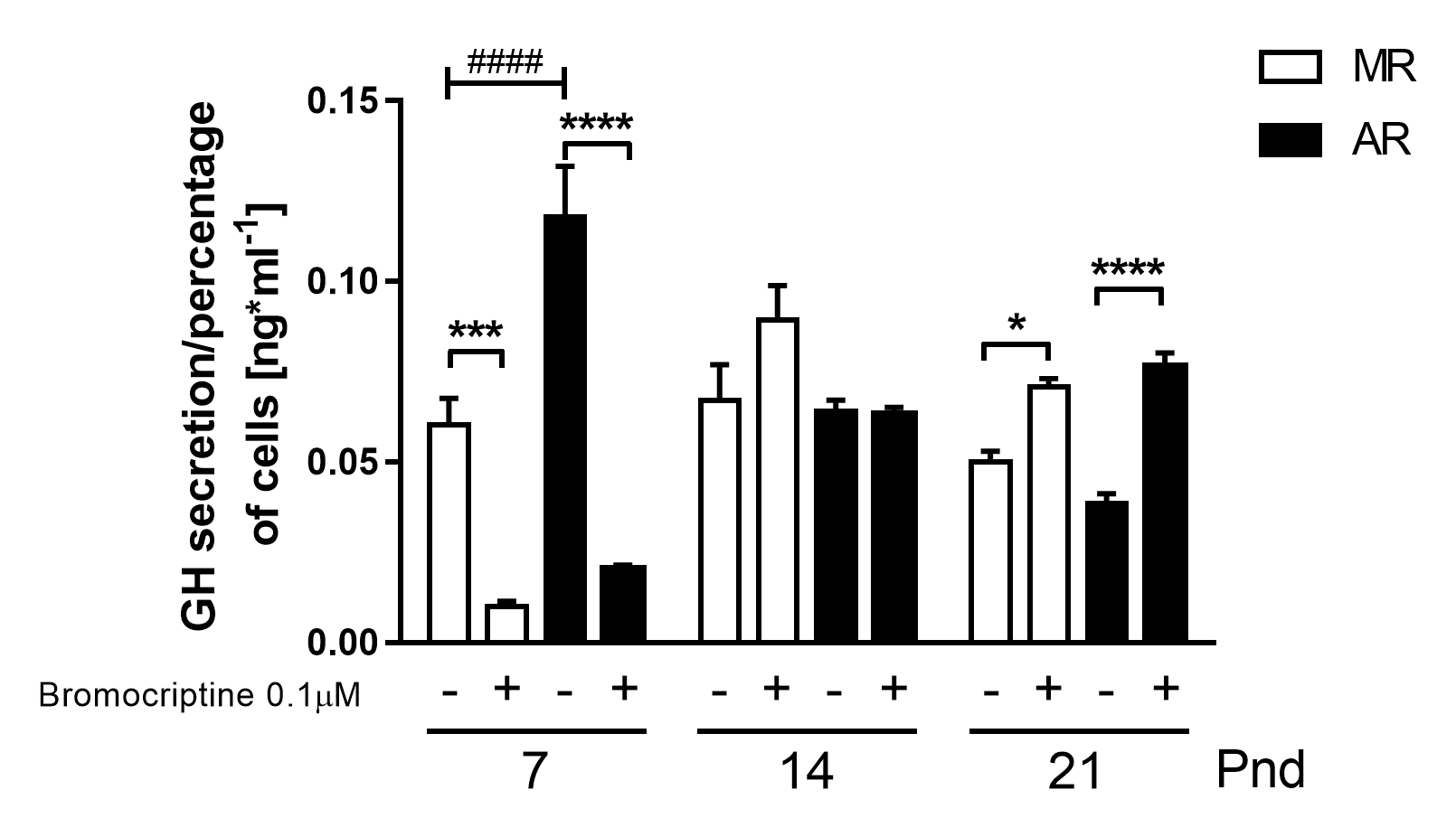

Supplement: S1 Fig — Cell cultures were performed with anterior pituitaries of MR and AR pups: at pnd7 (pools of 6 pups), pnd14 (pools of 3 pups) and at pnd21 (pools of 2 pups), n = 3 cell cultures. Basal and bromocriptine-stimulated GH-secretion were normalized by the percent of GH-positive cells. Differences between basal and bromocriptine GH secretion were analysed by two-way ANOVA, followed by Sidak´s multiple comparisons posttest for differences between MR and AR groups, #### P <0.0001. Statistical differences between MR and AR groups in GH secretion were analyzed by two way-ANOVA, followed by Sidak´s multiple comparisons posttest, * P <0.05, *** P <0.001, **** P <0.0001. (TIF) [file pone.0220853.s001.tif]
